# Complement anaphylatoxins C3a and C5a induce a failing regenerative program in cardiac resident cells. Evidence of a role for cardiac resident stem cells other than cardiomyocyte renewal

**DOI:** 10.1186/2193-1801-1-63

**Published:** 2012-12-12

**Authors:** David Lara-Astiaso, Alberto Izarra, Juan Camilo Estrada, Carmen Albo, Isabel Moscoso, Enrique Samper, Javier Moncayo, Abelardo Solano, Antonio Bernad, Antonio Díez-Juan

**Affiliations:** 1 Centro Nacional de Investigaciones Cardiovasculares Carlos III, Madrid, 28029 Spain; 2 Vascular Repair and Regeneration Laboratory, Centro de Investigaciones, Principe Felipe, Eduardo Primo Yúfera, Valencia, 46013 Spain

## Abstract

Cardiac healing, which follows myocardial infarction, is a complex process guided by intricate interactions among different components. Some resident cell populations with a potential role in cardiac healing have already been described in cardiac tissues. These non-cardiomyocyte cell subsets, globally described as cardiac pluripotent/progenitor cells (CPCs), are able to differentiate into all three major cardiac cell lineages (endothelial, smooth muscle and cardiomyocyte cells) in experimental settings. Nevertheless, physiological cardiac healing results in a fibrous scar, which remains to be fully modelled experimentally. Since a role for complement anaphylatoxins (C3a and C5a) has been described in several regeneration/repair processes, we examined the effects that C3a and C5a exert on a defined population of CPCs. We found that C3a and C5a are able to enhance CPC migration and proliferation. In vitro studies showed that this effect is linked to activation of telomerase mRNA and partial preservation of telomere length, in an NFκB-dependent manner. In addition, anaphylatoxin signalling modulates the CPC phenotype, increasing myofibroblast differentiation and reducing endothelial and cardiac gene expression. These findings may denote that C3a and C5a are able to maintain/increase the cardiac stem cell pool within the heart, whilst simultaneously facilitating and modulating resident cell differentiation. We found that this modulation was directed towards scar forming cells, which increased fibroblast/myofibroblast generation and suggests that both these anaphylatoxins could play a relevant role in the damage-coupled activation of resident cells, and regulation of the cardiac healing process after injury.

## Introduction

The cardiac healing process is guided by intricate interactions between different components; following myocardial infarction (MI), injury, inflammation, regeneration, and repair are all interconnected processes. It is known that these processes are inadequate and over-complicated, since certain pathological or damaging factors, such as cardiomyocyte replacement cannot be repaired. The inflammatory response represents one critical element of the cardiac healing process and is triggered by cell stress and death caused during cardiac injury. The abolition of the inflammatory response using corticosteroids not only decreases the number of infiltrating leukocytes, but also delays healing and collagen deposition (Kloner et al. 1978). Moreover it has been shown that cardiac tissue reperfusion improves overall tissue repair and that this outcome is mediated by improving the inflammatory reaction (Frangogiannis 2012).

Over the last 30 years, the complement system has been shown to play a major role in myocardial inflammation and tissue injury following MI (Hill and Ward 1971; Walport 2001) has been experimentally inhibited at different levels, including that of anaphylatoxin (C3a, C5a) signalling, to reduce ischemic injury. Experiments using animal models have shown that anaphylatoxin C5a inhibition protects against ischemia reperfusion (I/R) injury in diverse organs including myocardium (Vakeva et al. 1998). While anaphylatoxin C5a has been shown to be a potent activator of inflammation (Walport 2001), the upstream role of anaphylatoxin C3a has shown mixed results in animal models of I/R injury. In mouse models of renal and cerebral I/R injury, C3a appears to play a key role in mediating inflammation (Mocco et al. 2006; Thurman et al. 2007), whereas other studies suggest it is less important (Busche and Stahl 2010; Proctor et al. 2004). Moreover, in mammals, anaphylatoxins are critical for hepatocyte proliferation and liver regeneration (Strey et al. 2003). C3a promotes homing, chemotaxis, and retention of hematopoietic stem and progenitor cells in the bone marrow (Ratajczak et al. 2004); the anaphylatoxin receptors (C3aR, C5aR) positively regulate adult neurogenesis as well as the quantity of replacement neurons produced following cerebral ischemia (Rahpeymai et al. 2006). Recently, a novel and unexpected function for C3a and its receptor C3aR has also been revealed in the mutual cell-cell attraction (named co-attraction), required for maintaining cohesive clusters of migrating mesenchymal cells during early development (Carmona-Fontaine et al. 2011). The authors proposed that co-attraction and contact inhibition must act in concert to allow cell clusters to self-organise and respond efficiently to external signals, such as chemoattractants and repellents. Therefore anaphylatoxins seem to play both positive and negative roles depending on the physiopathological context; hence their artificial modulation to improve healing still requires further research.

The adult myocardium has recently been shown to harbour multipotent progenitor cells that can give rise to both myogenic and vasculogenic lineages, which can both contribute to myocardial repair (reviewed in (Barile et al. 2007; Laflamme and Murry 2011)). On the other hand, since the myocardium has a low endogenous regenerative competence, loss of a substantial amount of cardiac muscle ultimately results in scar formation. Inflammatory signals are required to guarantee the optimal creation of a supportive scar in the injured tissue, modulating phenotype function and gene expression in fibroblasts, endothelial cells, and leukocytes which together control collagen, fibroblast/myofibroblast deposition and vascular network formation. An optimal inflammatory reaction leads to stable scar formation, and the low regenerative potential of cardiac tissue indicates that, at least in a physiological situation, CPCs should behave preferentially as a healing cell population that participates in scar formation, since they have a very low potential for cardiomyocyte replacement. Telomerase-competent CPCs with long telomeres are present in the atria and apex storage regions of the heart; following activation by growth factors they migrate to damaged areas, where they although have the potential to create a population of young myocytes (Itzhaki-Alfia et al. 2009) their contribution to regenerated cardiac tissue is very low. Experimental data suggest that the regenerative potential of endogenous adult stem cells is low, and multiple reports show that defects in telomere maintenance impairs organ regeneration like liver (Hartmann et al. 2011) or in haematopoietic stem cell maintenance (Calado and Young 2008). In addition, telomere shortening during progenitor cell proliferation affects the function of the brain, pancreas, bone marrow, and heart, pointing to stem cell dysfunction as a critical determinant of organ aging/regeneration (Beausejour and Campisi 2006; Harrington and Greider 1991). Therefore efficient pro-regenerative signalling should modulate telomerase activation, allowing efficient cell proliferation, and also modulation of cell differentiation towards a healing phenotype.

In this work we describe the role of complement anaphylatoxins in CPC biology and prove that C3a and C5a are able to enhance cell migration and proliferation. This effect is linked to telomerase mRNA activity and activation, and a partial preservation of telomere length. Conversely anaphylatoxin stimulation influences CPC fate, pushing them towards myofibroblast differentiation, reduces endothelial gene expression, and increases collagen and smooth muscle gene expression, thus supporting a role for them in cardiac scar formation. Hence, enhanced proliferation ability and telomere length maintenance could denote that both C3a and C5a anaphylatoxins can help to maintain the cardiac resident cell pool within the heart during injury, and facilitate their function as scar forming cells.

## Results

### CPC culture and characterisation

**Figure 1:**
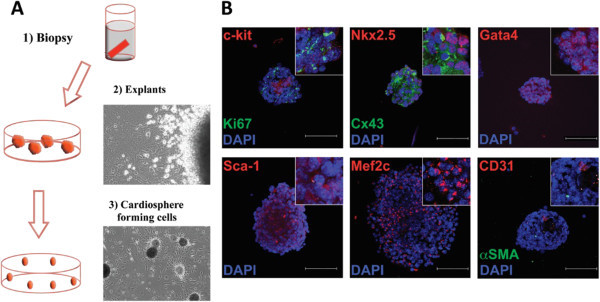
**Cardiosphere isolation and markers.A**) Cardiosphere isolation protocol. Cardiac explants are seeded in fibronectin-coated plates; after 2–3 days cells leave the explants and invade the plate where they start to proliferate. One week after, a population of loosely attached round bright cells appears, this population give rise to cardiospheres. **B**) Immunofluorescence analysis for cardiac, myofibroblast and endotheial markers in cultivated cardiospheres. Cardiospheres showed cells expresing c-kit and Sca-1. Presence of c-kit-positive cells are predominat in the core of the culture and relatively low in places that are positive for the proliferation marker Ki67. In contrast Sca-1+ cells are present uniformly in the whole culture. Cultures are also positive for Gata4, Cx43 and Mef2c cardiac markers. Expression of vascular markers, such as CD31 and SMA, was low.

Small biopsies of murine adult hearts were placed on gelatin/fibronectin plates (Figure 1A (1)). Following an initial outgrowth of fibroblast-like cells, within 5–7 days of explant plating, small, round and poorly adherent cells appeared and expanded (Figure 1A (2)). These cells, called explant-derived cells (EDCs) could be detached by gently pipetting, and were harvested and cultured to form cardiospheres (Figure 1A (3)). Using immunofluorescence, EDCs were found to express the cardiac markers, Nkx2.5, Gata4, Cx43, and Mef2c (Figure 1B) and also cell surface makers c-kit and Sca-1 (Figure 1B). During cardiosphere culture, proliferative cells (Ki67 positive) were found primarily in the external part of the sphere, in contrast to the CPC marker c-kit predominately found in the core. Sca-1 was more homogenously expressed throughout the whole culture. EDCs showed a very low expression for the vascular markers CD31 and aSMA.

**Figure 2:**
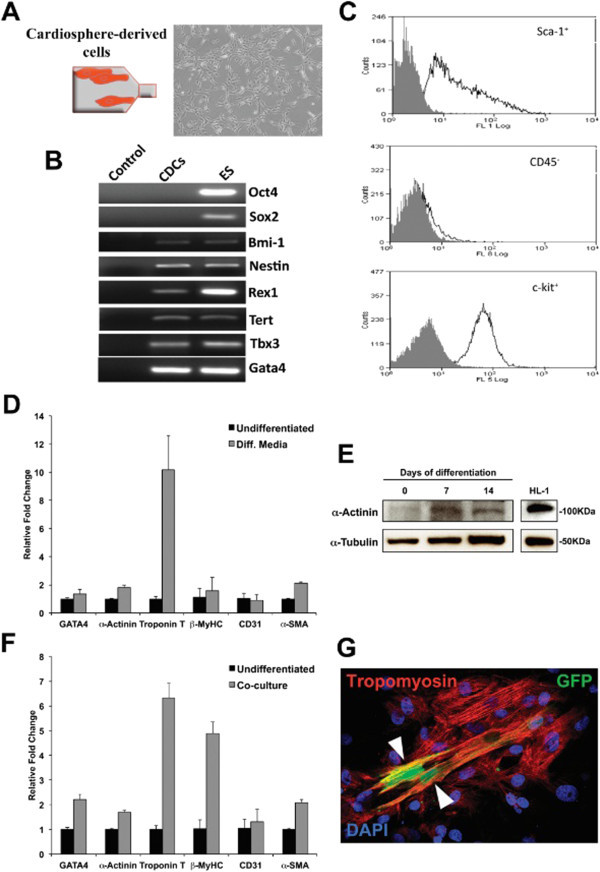
**CPCs isolation and differentiation.A**) CPCs are obtained when cardiospheres are expanded as a monolayer culture in fibronectin coated plates. **B**) mRNA expression of stemness (Oct4, Sox2, Bmi1, Nestin, Rex1, Tert) and early cardiac markers (Tbx3, Gata4). **C**) Flow cytometry showing the expression of Sca1 c-kit and CD45 in CPCs. **D**) Quantitative expression analysis of cardiac, endothelial and smooth muscle markers in CPCs exposed to differentiation medium. **E**) α-Actinin protein upregulation was confirmed by western-blot after 7 and 14 days in differentiation medium (HL-1 cells protein extract was used as a control). **F**) Quantitative expression analysis of cardiac, endothelial and smooth muscle markers in GFP^+^ CPCs after co-culture with NRCMs. **G**) Immunofluorescence showing GFP^+^ CPCs expressing Tropomyosin (white arrowheads) after 7 days in co-culture with NRCMs.

Cardiospheres were then expanded as a monolayer culture to passage 2 (p2); these cells, present within 3–4 weeks of biopsy, were termed multipotent cardiac progenitor cells (CPCs; Figure 2A). Flow cytometry on EDCs revealed that 84 ± 0.3% of these cells expressed the stem cell marker c-kit, 71 ± 9% expressed Sca-1 and 11 ± 2% expressed CD45 (Figure 2B) Gene expression was analysed using immunofluorescence and RT-PCR. CPCs expressed the pluripotent genes Bmi1, Nestin, Rex1, Tert, and lacked Oct4 and Sox2 expression (Figure 2B and some early cardiac transcription factors Tbx3, and Gata4. In order to evaluate their differentiation potential, CPCs were cultured with differentiation medium (Figure 2D and 2E) or co-cultured with neonatal rat cardiomyocytes (NRCMs; Figure 2F and 2G). After 7 days in differentiation medium culture, CPCs showed upregulation of cardiac genes such as Troponin T and α-Actinin (Figure 2D), which was confirmed by western-blot (Figure 2E). GFP^+^ CPCs were co-cultured with NRCMs and after 7 days isolated by FACS for molecular analysis. Gene expression studies showed upregulation of Gata4, α-Actinin, Troponin T, β-MyHC and α-SMA (Figure 2F) and immunofluorescence analysis revealed the presence of GFP^+^ CPCs derived cells expressing Tropomyosin (Figure 2G).

### CPCs express functional anaphylatoxin receptors

**Figure 3:**
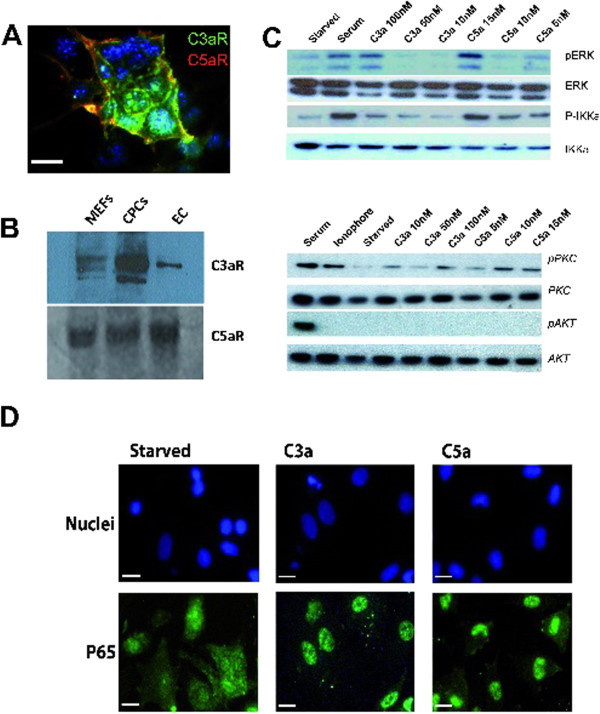
**CPCs possess functional complement receptors C3aR and C5aR that signal through different pathways.** (**A**) Immunoflourescence de C3aR (FITC) and C5aR(Cy5) on CPCs culture. **B**) Immunoblot of CPCs to detect C3aR and C5aR receptors. **C**) Immunoblot of CPCs stimulated with different concentrations of C3a and C5a. C3a C5a stimuli activate PKC, ERK1/2 and NFκB pathways but fail to activate AKT. ERK, AKT and PKC activation are measured as presence phosphorylated forms. NFκB activation is probed as IKK phosphorylation. **D**) p65 nuclear translocation. Starved CPCs were induced with 100nM C3a or 15 nM C5a, Immunoflorescence of p65 protein reveal nuclear translocation. Serum stimulation is the positive control for activation ERK, AKT and NFkappa Beta. Calcium ionophore is the positive control for PKC activation. MEFS = mouse embryonic fibroblasts, EC = Murine endothelial cell line. IgG Anti C3aR-FITC (green). IgG Anti C5aR-Texas (Red). White bar: 10 μm.

Functional expression of the anaphylatoxin receptors C3aR and C5aR on non-immune cells has been previously reported for other cells like neurons and astrocytes (Van Beek et al. 2000) or mesenchymal stem cells (Schraufstatter et al. 2009), between others. To evaluate the potential of complement anaphylatoxins to activate CPCs, we checked the expression of C3a and C5a receptors on CPCs. We detected cell surface expression of C3aR and C5aR determined by immunofluorescence (Figure 3A), which was confirmed by Western blotting for their respective proteins (Figure 3B). The entire cell population expressed cell surface C3aR and C5aR in moderate to high levels. Interestingly, densitometry analysis of western blot membrane and normalized the expression versus C5aR the expression of C3aR in CPCs was 7,9 fold higher than in endothelial cells (ECs) or 2,5 fold higher compared to mouse embryonic fibroblasts (MEFs). To probe the functionality of the C3a and C5a receptors, we explored the activation of signalling pathways known to be triggered by anaphylatoxins in other cell types. These pathways are the G-protein calcium-release dependent phosphorylation of PKC, and also the phosphorylation-dependent activation of ERK1/2 (Monsinjon et al. 2003), AKT/PKB (Schraufstatter et al. 2009), and NFkB (Pan 1998). We found substantial activation of both the ERK and PKC pathways, but we did not detect the phosphorylated forms of Akt (Figure 3C). In addition C3a, and to a larger extent C5a, were able to induce NFκB activation, measured as the phosphorylation of IKKa (Figure 3C) and the nuclear translocation of p65 (Figure 3D).

### C3a and C5a promote CPC proliferation and migration

**Figure 4:**
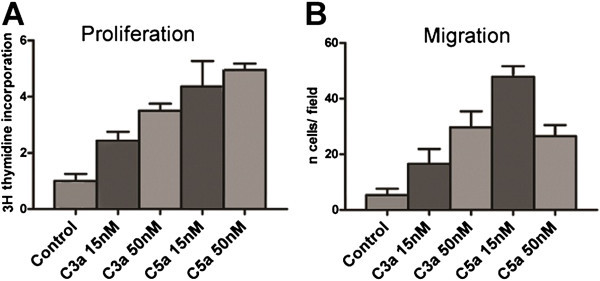
**C3a**/**C5a induce proliferation and migration in CPCs.A**) CPCs treated with C3a and C5a showed an increase in DNA 3H-thymidine incorporation with respect to the control. **B**) CPCs migrated towards C3a and C5a gradients when migration was assessed in a Boiden chamber assay. The optimal doses for both proliferation and migration are C3a = 50 nM and C5a = 15 nM. Data were normalized versus control. p ≤ 0.05 (*); p ≤ 0.01 (**).

Proliferation and migration to damaged tissues are two important characteristics of progenitor cells that enable them to perform their physiological role in maintaining tissue homeostasis. In the context of cardiac homeostasis, the release of complement anaphylatoxins is a signal of tissue damage, which in turn may have an effect on any cardiac progenitors close to the damaged area. To probe this hypothesis we assessed the proliferative and migration ability of CPCs upon C3a and C5a stimulation (Figure 4A). CPCs were cultured in the presence of C3a and C5a, and their proliferation rate was measured by 3H-thymidine incorporation into their DNA. CPCs cultured in the presence of C3a and C5a proliferated faster than the control cells. This proliferation enhancement was dose dependent and peaked at C3a = 50 nM (3-fold vs. control), and C5a = 15 nM (4-fold vs. control; Figure 4A). Moreover effect of C5a is quite more stronger to activate migration and proliferation than C3a. The addition of both anaphylatoxins at the described peak concentration together the effect of C5a overlaps C3a with out any cooperation.

In some models cell proliferation is linked to cell migration (Diez-Juan and Andres 2003). Interestingly, in addition to the modulation of the chemotactic activity of immune cells, C3a and C5a has also been shown to induce migration of non-immune cells such as neural stem cells (Shinjyo et al. 2009) or mesenchymal stem cells (Schraufstatter et al. 2009), between others. Therefore we next explored the potential of C3a and C5a as chemotactic factors in CPCs. Using a Boyden chamber, CPCs were placed in the upper transwell compartment and stimulated with C3a or C5a in the lower compartment (Figure 4B). Both anaphylatoxins were able to induce cell migration when present at the same concentration that induced cell proliferation in the previous assay. C5a was more efficient at inducing cell migration at lower doses; at higher concentrations the stimulation was reduced, resulting in a bell-shaped response curve (Figure 4B), which may be explained by the fact that this agent primarily stimulates chemotaxis. In fact, at higher concentrations, diffusion of C5a from the lower to the upper compartment of the Boyden chamber could disrupt the C5a gradient and thus prevent chemotactic migration. In contrast, the use of a chemoattractant that stimulates chemokinesis would not result in a bell-shaped dose–response, since such a reaction is independent of chemical gradients.

### Anaphylatoxins C3a and C5a induce telomerase activity and telomere maintenance in CPCs

It is well known that cell proliferation is dependent on telomere maintenance, which is mainly achieved through the action of telomerase (Tert) (Choudhary et al. 2012). Moreover, multiple experimental data show that telomere length maintenance is crucial to the preservation of the regenerative potential of adult endogenous stem cells (reviewed in (Choudhary et al. 2012)). Thus, we assessed if C3a and C5a could be considered as a pro-healing stimuli by activating CPC proliferation in combination with telomerase induction.

**Figure 5:**
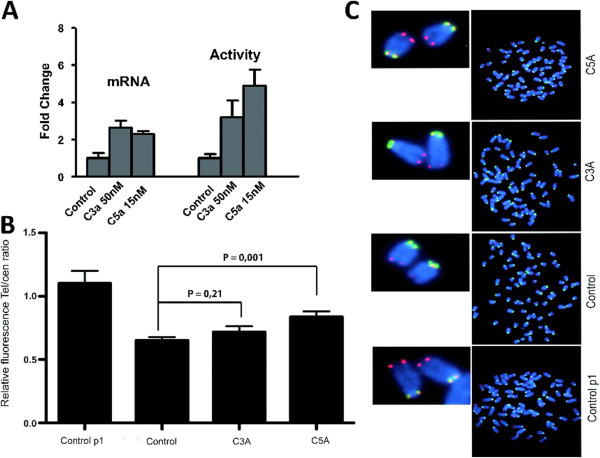
**Telomerase analysis.A**) Telomerase mRNA expression and TRAP activity in CPCs cultured in presence of C3a or C5a. B and C) CPCs cultured in presence of C5a and C3a present longer telomeres than their counterparts. **B**) Quantification of relative fluorescence of telomere vs. centromere FISH probe Three independent experiments was analyzed. p ≤ 0.05 (*); p ≤ 0.01 (**). **C**) Representative figures of chromosome FISH. Centromere (red) Telomere (green).

To test this idea, we measured telomerase expression and activity in CPCs cultured in the presence of C3a or C5a at the optimal doses of 50 nM and 15 nM respectively (Figure 5A). We found, by qPCR analysis, that CPCs cultured with C3a and C5a showed more than a 2-fold increase in Tert mRNA (Figure 5A). This result was confirmed by measuring telomerase activity using a telomeric amplification protocol (TRAP). CPCs cultured in the presence of C3a and C5a showed an incremental increase in telomerase activity of 3-fold and 5-fold respectively, with respect to the control cells (Figure 5A). We next examined whether the pro-telomeric role of C3a and C5a anaphylatoxins is correlated to the presence of longer telomeres than in controls. We used a telomeric probe, normalised with a centromeric probe, to quantify the telomere length of CPCs grown in presence of C3a or C5a for 20 population doublings. As shown in Figure 5B and C, C3a-CPCs and C5a-CPCs display longer telomeres than their control counterparts. However, this telomere maintenance effect is not capable of completely preventing telomere shortening, since C5a-CPCs at p20 have still significantly shorter telomeres than their counterparts at p0. In addition, this increment was only statistically significant (P = 0.001) in the C5a treated CPCs (Figure 5B). Taken together, these results suggest that C3a and C5a have a ‘pro-telomeric’ effect on CPCs, although this is not sufficient to retain telomere length at their initial length.

### The induction of TERT expression by anaphylatoxins C3a and C5a is dependent of NFκB activation

To gain insight into the molecular mechanisms responsible for C3a and C5a anaphylatoxin mediated Tert induction we examined the Tert promoter and found two NFκB binding sites. Since C3a and C5a are able to induce NFκB signalling in CPCs, this molecular pathway appeared to be a good candidate for the mediation of C3a and C5a dependent Tert mRNA expression.

**Figure 6:**
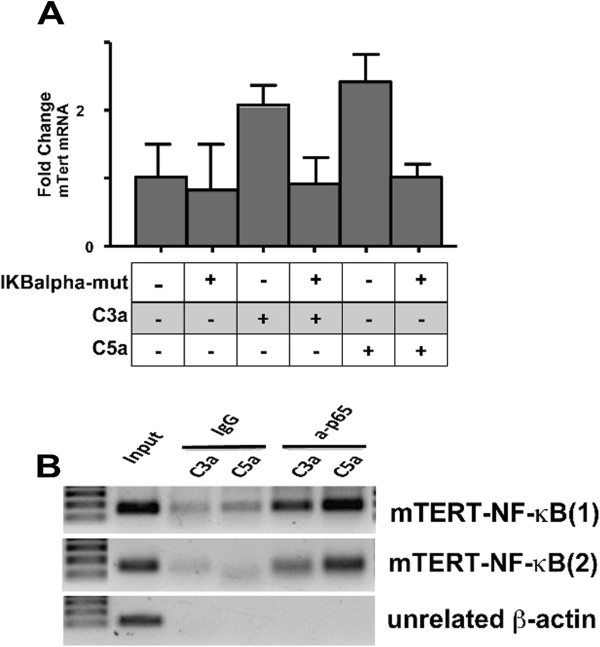
**NFκB signalling mediates the induction of Tert by C3a and C5a.A**) CPCs transduced with a retroviral vector coding for mutant IκBα that blocks NFκB activation or with a control vector and induction of Tert mRNA expression was assessed by RT-qPCR. **B**) Chromatin immunoprecipitation (CHIP) of the TERT promoter using a p65 antibody. Input (complete extract), IgG (IgG isotipe control), a-p65 (IP of chromatin binding sites using p65 antibody). Two different primers at the NFκB site of Tert promoter were used mTert-NF-κB(1) and mTert-NF-κB(2). ß-actin primers were used as a control.

To test this possibility, we used two complementary approaches. First, we performed a chromatin immunoprecipitation assay for the NFκB subunit, p65; we used primer pairs that cover the NFκB site at −326-316 in the murine TERT promoter so that we could determine if p65 is recruited to the endogenous TERT promoter upon anaphylatoxin stimulation. These CHiP assays confirmed that C3a/C5a stimulation induces the recruitment of p65 to the consensus site in the murine TERT promoter (Figure 6B). Second, to further confirm that NFκB was responsible, at least in part, for inducing TERT mRNA expression in anaphylatoxin-stimulated CPCs, we used a mutant of IκBα protein that is refractory to IKK phosphorylation. This mutant IκBα S32/S36-A behaves as a potent dominant negative IκBα protein that attenuates NFκB transactivation (Brown et al. 1995). CPCs were transduced with a retroviral vector coding for IκBα to block NFκB activation or with a control vector, and Tert mRNA expression was assessed by RT-qPCR. As shown in Figure 6A, attenuation of NFκB activation abolishes anaphylatoxin dependent Tert induction in CPCs. Taken together these experiments demonstrate that NFκB is the main signalling pathway involved in anaphylatoxin dependent Tert expression.

### Anaphylatoxins C3a and C5a abolish the cardiac and endothelial potential of CPCs and promote myofibroblastic differentiation mediated by an endothelial to mesenchymal transition-like process

Although some experimental results have shown a limited regenerative response after heart injury, cardiac wound healing in mammals is hampered by the fact that the regeneration of heart muscle is virtually absent, and that damaged myocardium is replaced by scar tissue. The above results point towards pro-regenerative activity for complement anaphylatoxins: both of them promote CPC migration, proliferation, telomerase activity, and telomere maintenance. All of these anaphylatoxin-mediated processes can be envisioned to activate and expand the resident CPC pool within the heart. However, to be effective in regenerating damaged tissue, a stem cell pool must preserve its differentiation potential when it proliferates. A pro-regenerative event would mean that CPCs maintain their ability to differentiate into the main cardiogenic lineages (smooth muscle/myofibroblast, endothelium and cardiomyocytes), or are even able to enhance their ability to differentiate towards a cardiomyocyte fate, the main cell type that is not replenished after MI. Therefore we examined the effect of CPC exposure to complement anaphylatoxins.

**Figure 7:**
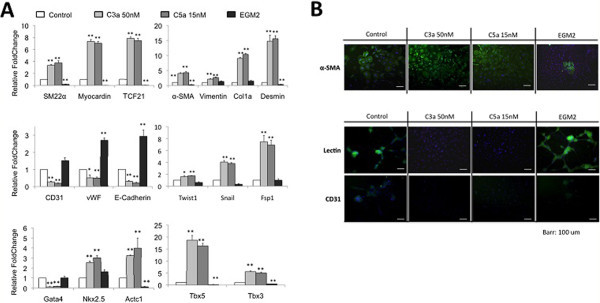
**Effects of complement anaphylatoxins upon stemness myofibroblast and cardiogenic potential of CPCs.A**) C3a and C5a induces expression of miofibroblast transcription factors (SM22a, myocardin, TCF21, α-SMA) and markers (Vimentin, Collagen and Desmin). In other hand reduces expression of endothelial genes (CD31, vWF and E-Cadherin). Genes involved in endMT (Twist1, Snail and Fsp1) are also induced. There is an increase in cardiogenic genes as NKx2.5, Actc1, Tbx5 and Tbx3. But Gata4 is repressed. **B**) Complement anaphylatoxins induce myofibroblast differentiation. Treatment with C3a and C5a produces a marked increase in the mRNA levels of core factors involved in myofibroblast differentiation (Myocardin, SM22alpha, TCF21) as well as in the mRNA levels of the mature myofibroblast markers SM-actin, Desmin, Vimentin, and Col1a. **B**) Immunofluorescence confirms data presented in B; CPCs treated with C3a or C5a present higher number of cells positive for SMA. As a positive control for endothelial differentiation, CPCs were cultured in Endothelial Media (EGM); in **A**) cells cultured in this condition repress the expression of myofibroblast and endMT associated genes and induce the expression of the endothelial markers. p ≤ 0.05 (*); p ≤ 0.01 (**).

CPCs treated with either C3a or C5a (for 72 h) were assessed, by RT-qPCR, for the expression of some well-known endothelial, myofibroblast, and cardiomyocyte lineage markers. We chose Gata4, Nkx2.5, Tbx5, and Tbx3 as cardiogenic markers, Actc1 as an early cardiomyocyte marker, CD31, vWF, and E-Cadherin as endothelial markers, Myocardin, SM22α, and TCF21 as myofibroblast markers, and SM22α, α-SMA, Vimentin, Desmin, and Collagen 1a (Col1a) as mature myofibroblast markers. We found that upon anaphylatoxin stimulation, CPCs appeared to initiate a myofibroblastic transcription program, with a more than 5-fold increase in the pro-myofibroblastic transcription factors Myocardin and TCF21. In addition, there was a clear increase in the expression of the myofibroblast markers Desmin, α-SMA, Col1a, and Vimentin, which clearly indicates differentiation towards a mature myofibroblast fate (Figure 7A). The induction of myofibroblast genes was accompanied by a reduction of endothelial markers with more than a 3-fold decrease in the quantity of CD31 and E-Cadherin mRNA detected, and a 2-fold decrease in the case of vWF. It therefore seemed clear that anaphylatoxin stimulation compromises the endothelial fate of CPCs and promotes a clear bias towards the myofibroblastic fate. This was further confirmed by immunofluorescence analysis of the myofibroblast marker α-SMA, and of two endothelial markers CD31, and the surface protein lectin. As shown in Figure 6B, CPCs treated with C3a or C5a showed increased levels of α-SMA positive cells, typical of myofibroblastic lineages. Conversely, CPCs stimulated with C3a or C5a lost CD31expression and showed a reduction in tomato-lectin binding on their membranes. As a positive control, we cultured CPCs in endothelial medium (EGM2) to direct endothelial differentiation. At the mRNA level, this resulted in a 2.5-3-fold increase in endothelial marker levels and the abolition of myofibroblast markers (Figure 7A). Immunofluorescence studies revealed that, with respect to the control, EGM2 cultured CPCs presented higher levels of lectin and slightly increased levels of CD31 (Figure 7B); this mirrors the small increase in CD31 mRNA expression in EGM2 cultured CPCs with respect to control CPCs.

Immunofluorescence analysis showed that when CPCs are in their multipotential state, they express moderate levels of both myofibroblast and endothelial markers (Figure 7) and so they can be considered as being in a state of balance between these two fates. Complement anaphylatoxin stimulation seems to break this balance, pushing CPCs towards the myofibroblast lineage. We then hypothesized about the mechanism underlying this C3a/C5a-dependent balance shift and noticed that the decrease in the endothelial markers could point to an endothelial to mesenchymal like transition (EndTM), which would be capable of altering this equilibrium. To test this hypothesis, we treated CPCs with the optimal C3a and C5a doses and measured the mRNA levels of two key transcription factors involved in EndMT, Snail1 and Twist1 (Huber et al. 2005). Figure 7A shows that cells treated with C3a and C5a presented higher levels of Twist1 (almost 2-fold) and Snail (> 5-fold) mRNAs. We also assessed the expression of the known EndMT marker Fsp1 and confirmed the C3a/C5a-dependent induction of an EndMT-like process; CPCs treated with these anaphylatoxins showed a 5-fold increase in the Fsp1 mRNA levels (Figure 7A).

Therefore a likely explanation for the observed results is that C3a and C5a promote an EndMT-like process in CPCs that restricts their endothelial potential and pushes them towards the mesenchymal fate inducing the expression of the myofibroblastic lineage promoters and thus committing them to differentiate towards myofibroblasts that express the typical markers Desmin, α-SMA and, SM22α. In addition, the cardiogenic potential of CPCs is abolished upon C3a and C5a stimulation, since this negates C3a and C5a dependent commitment of CPCs into myofibroblasts. Expression of the key pioneer transcription factor in cardiogenesis, Gata4, was clearly repressed upon anaphylatoxin treatment (Figure 7A); however the expression of other cardiogenic factors did not follow this pattern. When CPCs were treated with C3a and C5a, we found that while Gata4 mRNA decreased 5-fold, the mRNA levels of its downstream factors Nkx2.5, Tbx5 and Tbx3 markedly increased (2.6 to 17-fold), along with an increase in mRNA from the early cardiac marker Actc1, which rose by 3 - 4-fold.

Gata4 is known to be crucial for cardiomyocyte differentiation; therefore, the repression of Gata4 expression in CPCs stimulated by C3a and C5a clearly points toward a role for these factors in the compromise of cardiogenic differentiation potential in these CPCs. A role for C3a and C5a in the compromise of cardiogenic differentiation is further supported by the appearance α-SMA positive cells and by the induction of myofibroblast markers in C3a/C5a treated CPCs, since this process is obstructing cardiomyocyte differentiation (Wu et al. 2006). Therefore the C3a and C5a mediated transcriptional activation of Tbx3, Tbx5 and Nkx2.5 might suggest an alternative role for these transcription factors in the maturation of and/or functional properties of myofibroblasts. Another interesting possibility is the implication of these mesodermal factors in the C3a/C5a mediated proliferation of CPCs.

Thus, we conclude that C3a and C5a promote the differentiation and commitment of CPCs towards myofibroblast lineage, blocking their cardiac and endothelial potential.

## Methods

### Cell culture and stimulation

Sca1^+^ CPCs were grown on gelatin at 0.1% (v/v) in CSPH Media, composed of 50% Neurobasal Medium, 50% DMEM/F12, 1% 100 μg/ml penicillin and streptomycin, 1% 2 mM L-Glutamine, 0.1% 250 ng/ml Fungizone, 0.1% 50 mg/ml Gentamicin and 10% Stem Cell Quality (ESQ)-FSB, supplemented with LIF 10 ng/ml, 1% ITS, FGF 10 ng/ml, EGF 20 ng/ml, 1% N2 and 2% B27. C3a (FIDCCNH ITKLREQHRR DHVLGLAR) and C5a (AFNECCTIAN KIRKESPHKP VQLGR) synthetic peptides were synthetised by GeneScript USA, NJ. Inmortalized endothelial cell line was a gift of Dr. Alfonso Luque and has been previously described (Hortelano et al. 2010). Mouse embryonic fibroblasts (MEFs) were generated from mice littermates in C57BL/6 J background harvested from embryonic day12-13.5. Briefly, separated embryos from pregnant mice had their heads and livers removed and incubated at 37°C in the presence of 0.25% Trypsin/EDTA for 5 minutes in a humidified incubator. Trypsinized embryos were homogenized by pipetting up and down and allowed to sediment for 5 minutes, and cells sedimenting in the lower phase were collected and plated in fresh DMEM containing 10% FBS. If not specified culture mediums and supplements were obtained from Invitrogen, (Carlsbad, CA). Growth factors were purchased from Peprotech (Oak Park, CA)

### Differentiation assays

CPCs were exposed to differentiation medium for 7 and 14 days, composed of DMEM, 1% 100 μg/ml penicillin and streptomycin, 1% 2 mM L-Glutamine, 10% Stem Cell Quality (ESQ)-FSB and 10nM Dexamethasone. Neonatal rat cardiomyocytes were isolated from P2 Wistar rat hearts using the Worthington Neonatal Cardiomyocyte Isolation System (Worthington, Lakewood, NJ). CPCs for co-culture experiments were transduced with pRRL.CMV.GFP lentiviral vector supernatants provided by CNIC viral vector facility, and cells with high GFP expression were selected by FACS. For the co-culture, NRCMs were seeded at 7.5 × 10^4^ cells/cm^2^ on 75% M199, 25% DMEM, 5% FBS, 10% HS, 1% 100 μg/ml penicillin and streptomycin and 1% 2 mM L-Glutamine, and on the next day GFP^+^ CPCs were added at 3 × 10^3^ cells/cm^2^.

### Proliferation assay

Proliferation was measured by [3H]thymidine incorporation performed in triplicate as previously described (Diez-Juan and Andres 2003). Briefly cell cultures were plated on 24-well culture dishes at a density of 5 × 10^3^ cells per well and incubated for 4 hours with [3H]thymidine (1 μCi/mL, Amersham) after which cells were washed with PBS and incubated for 1 h with ice-cold 5% trichloroacetic acid. Labeled DNA was extracted with 0.5 N NaOH and counted in a scintillation counter (Wallac, Turku, Finland).

### Migration assay

CPCs were plated at 1 × 10^5^ cells/well in the upper well of a transwell (8 μm pore size, Millipore) in 250 μl of serum free DMEM/F12 media supplemented with ITS (Invitrogen, CA, USA). Cells were allowed to migrate towards the lower transwell compartment (500 μl chemotactic gradient (C3a 50 nM/C5a 15 nM) for 3 hours at 37°C. CPCs were mechanically removed from the top side of the transwells and the membranes were fixed for 15 min with 4% PFA, stained with Prolong DAPI (Invitrogen, CA, USA) and counted. Experiments were performed in triplicate.

### Immunofluorescence

Cells were cultured in plastic tissue cover slips and fixed with 2% PFA. Cells were then washed twice with PBS, blocked with 10% horse serum and incubated with antibodies overnight. Specific fluorescent secondary antibodies were use to visualise immunolabelling. We used anti- Tropomyosin (Sigma), anti-C5aR/cd88 (AM01269PU-S, Acris Gmbh), anti-C3aR (sc-14624, Santa Cruz), anti-α-SMA (1A4, A2547, Sigma) and CD31 (MEC13.3, BD Pharmigen). Biotinilated anti goat IgG (Invitrogen) was added after 3 washes with PBS-T. Finally anti mouse FITC or Cy3 streptavidin (Invitrogen) was added.

### Western blots

Cells were lysated with RIPA buffer. The protein concentration was quantified by Protein detection kit "DC protein assay" (BIO-RAD, Hercules, CA, US), and 10 μg of protein was loaded into a 12% acrilamide gel. The proteins were transferred with iBlot Dry Blotting System (Invitrogen), and the membranes were blocked with 10% dry milk in PBS-T. The membranes were then incubated overnight at 4°C with the primary antibody in blocking buffer. This was followed by washing and 1 hour incubation with an appropriate peroxidase-conjugated secondary antibody. Protein bands were detected by chemiluminiscence using a commercial kit (Pierce) according to the manufacturer’s instructions. Primary antibodies were: anti p65, anti iKKa, anti p-IKKa anti pERK, anti ERK1/2, anti pPKC, anti PKC, anti p-AKT anti and AKT from Cell Signaling; rat IgG (Millipore), anti α-Actinin murine IgG (Sigma), anti Tubulin murine IgG (BD biosciences), anti C3aR Antibody (H-300) sc-20138 and anti C5aR(CD88) (H-100) sc-25774 (Santa Cruz Biotechnology). Secondary antibodies were HRP-anti rat IgG (Dako) and HRP-anti mouse IgG (Dako). If stated, densitometric analysis of the blots was performed with ImageJ software.

### RNA extraction and nonQuantitative and quantitative real-time polymerase chain reaction (RTq-PCR)

Total RNA was extracted using the PureLink RNA Mini Kit (Invitrogen). First strand cDNA was synthesised using the High Capacity cDNA Reverse Transcription kit and random hexamers (Applied Biosystems). For RTq-PCR, samples were run in 20 μl reactions using an ABI 7900 Fast (Applied Biosystems, Foster City, CA). Samples were incubated at 95°C for 15 min, followed by 40 cycles at 95°C for 10 s, 60°C for 20 s, and 72°C for 30 s. SYBR Green oligonucleotides were used for detection and quantification of a given gene. The relative mRNA level was calculated after normalization to two standard housekeeping genes (36B4 and GusB) using the Comparative Ct Method as described in the manufacturer’s instructions (Invitrogen, Carlsbad, CA). For nonQuantitative, PCR samples were run in 15 μl reactions contained cDNA, 0.2 mM dNTP, 0.5 μM 5 and 3 oligonucleotide primers as well as reaction buffer and 1.25 U of DNA polymerase (HotStarTaq; Qiagen, Hilden, Germany). DNA amplification was conducted using a thermocycler (Mastercycler; Eppendorf, Hamburg, Germany). PCR conditions were 28 cycles of 94°C (1 min), 59°C (1 min), and 72°C (1 min) Primers are described in Table 1 and were designed using bioinformatic methods and checked for specificity by running the final reaction in a 2% agarose gel as well as by obtaining their corresponding melting curves.

**Table 1:** **Primer sequences**

Primer Name	SEQUENCE
36B4 Fw	AGATGCAGCAGATCCGCAT
36B4 Rev	GTTCTGCCCATCAGCACC
Actc1Fw	GCTCTGGGCTTCATCACCTA
Actc1Rev	AGCTGTCTTCCCGTCCATC
β-MyHC Fw	GAGCCTTGGATTCTCAAACG
β-MyHC Rev	GTGGCTCCGAGAAAGGAAG
CD31 Fw	AGTTGCTGCCCATTCATCAC
CD31 Rv	CTGGTGCTCTATGCAAGCCT
Col1a1Fw	TAGGCCATTGTGTATGCAGC
Col1a1Rev	ACATGTTCAGCTTTGTGGACC
Desmin Fw	TACACCTGCGAGATTGATGC
Desmin Rev	ACATCCAAGGCCATCTTCAC
E-Cadherin Fw	GAGGTCTACACCTTCCCGGT
E-Cadherin Rev	AAAAGAAGGCTGTCCTTGGC
Fsp1 Fw	TCAGCACTTCCTCTCTCTTGG
Fsp1 Rev	AACTTGTCACCCTCTTTGCC
Gata4 Fw	CCATCTCGCCTCCAGAGT
Gata4 Rev	CTGGAAGACACCCCAATCTC
Gusb Fw	ACTCCTCACTGAACATGCGA
Gusb Rev	ATAAGACGCATCAGAAGCCG
Myocardin Fw	TTAAGCCTTGGTTAGCCAGC
Myocardin Rev	GGGGTCTGAACACTCTTTGC
Nkx2.5 Fw	GGCTTTGTCCAGCTCCACT
Nkx2.5 Rev	CATTTTACCCGGGAGCCTAC
SM 22 alpha Fw	CCTCCAGCTCCTCGTCATAC
SM 22 alpha Rv	CCTTCCAGTCCACAAACGAC
SM-actin Fw	TCCCTGGAGAAGAGCTACGAACT
SM-actin Rev	GATGCCGCTGACTCCAT
Snail Fw	TCC AAA CCC ACT CGG ATG TGA AGA
Snail Rev	TTG GTG CTT GTG GAG CAA GGA CAT
Tbx3 Fw	CATTGCCAGTGTCTCGAAAA
Tbx3 Rev	TCCCGGAAACAGAATTCATC
Tbx5 Fw	TGGTTGGAGGTGACTTTGTG
Tbx5 Rev	GGCAGTGATGACCTGGAGTT
Tcf21 Fw	GCAGATCCTGGCCAACGACA
Tcf21 Rev	CGGTCACCACTTCCTTCAGGTCA
Troponin T Fw	ACCCTCAGGCTCAGGTTCA
Troponin T Rev	GTGTGCAGTCCCTGTTCAGA
Twist1 F	AAT TCA CAA GAA TCA GGG CGT GGG
Twist1 R	TCT ATC AGA ATG CAG AGG TGT GGG
Vimentin Fw	TCCACTTTCCGTTCAAGGTC
Vimentin Rev	AGAGAGAGGAAGCCGAAAGC
vWF Fw	CTCACACAGAGCCACAAAGG

### Telomere length quantification by Q-FISH

FISH was carried out as described above, using a Cy3- labelled LL(CCCTAA)3 peptide nucleic acid (PNA) telomeric probe (Euregentec) and a FITC labelled LL(ATTCGTTGGAAACGGGA) PNA alpha satellite probe (Eurogentec, Liège) as previously described (Samper et al. 2001) with the following modifications. After hybridisation slides were washed three times with PBS supplemented with 0.1% Tween for 10 min at 60°C and dehydrated through an ethanol series (70%, 90%, and 100%) for 5 min each. Slides were then counterstained and mounted in Vectashield H-1200 mounting medium. Digital images were acquired as described above. Telomere signals were captured with the same exposure time in all samples and the telomere length was represented in relative fluorescence units. Telomere signals from at least 20–30 nuclei per group were quantified using the TFL-Telo (version 2) kindly provided by Dr Peter Lansdorp (British Columbia Cancer Centre, Vancouver). All images were captured and analysed in parallel on the same day by an experimenter blinded to the treatment groups.

### Measurement of telomerase activity by TRAP assay

Telomerase assays were performed on 5000 CPCs as described (Banerjee and Jagadeesh 2009), with the following modifications. Protein extracts were made using lysis buffer NP40 (10 mM Tris–HCl, 1 mM MgCl2, 1 mM EDTA, 1%NP40, 0.25 mM Sodium deoxycholate, 10% glycerol, 150 mM NaCl, 5 mM β-mercaptoethanol (all from Sigma-Aldrich) and protease inhibitor 1x (Roche, Basel, Switzerland) at pH 8.0. The protein concentration was quantified by Protein detection kit "DC protein assay" (BIO-RAD, Hercules, CA, US). Were incubated 5 and 1 μg of protein per sample with telomerase extension buffer (500 mM Tris-AcH, 500 Mm AcK. 30 mM MgCl2, 10 mM spermin, 10 mM EGTA, 50 mM β-mercaptoethanol, 2 mM dAGT (all from Sigma-Aldrich) and 1 mM of Oligo TS (5´ - AAT CCG TCG AGC AGA GTT −3`. Extension reaction of telomerase (2 μl) was added to 23 μl PCR reaction mix containing 1 x Power SYBR Green PCR Master Mix, 5 mM EGTA, 2 ng/μl Oligo TS and 4 ng/μl Oligo ACX (5´ GCG CGG C(TTACCC)4. PCR was carried out at 94°C for 10 min followed by 40 cycles of, 94°C for 15 s and 60°C for 1 min. PCR products were monitored with a ABI PRISM 7700 sequence detection apparatus (Applied Biosystem, Foster City, CA, USA) and analyzed with SDS v2.3 software (Applied Biosystem, CA).

### Chromatin immunoprecipitation assays

Chromatin immunoprecipitation (ChIP) experiments were performed using the EZ-Magna ChIP G kit (Millipore Corporation), according to the manufacturer's protocol, with slight modifications. Approximately 107 CPCs were used for each experiment. Two millilitres of cell lysate were sonicated using a Microson Ultrasonic Cell Disruptor. Samples were kept cool in an ethanol-ice-water bath and sonicated for four 30 s pulses with 1 min pauses between them. Fragmented chromatin was diluted in ChIP dilution buffer (Millipore EZ-Magna ChIP kit), distributed in 300 μl aliquots, and used directly or stored at −80°C. The anti-p65 (ab31481) and anti-histone H3 (ab1791) antibodies were purchased from Abcam. Following immunoprecipitation, DNA was analysed by real-time PCR. To identify NFκB binding sites, 3Kb upstream of murine Tert gene were analyzed using PROMO (Messeguer et al. 2002). One site was found at position chr13:73764072–73764172 at -322 bp of mTert star site within dissimilarity margin less or equal than 5%. The following primers were used TertFw1: GCCCAAACCTCGCCCCAGTC, TertFw2: CCCAAACCTCGCCCCAGTCT, TertRw1: TGCTTCTCGGGCTTCCTTTTCGC, TertRev2: ACAGAATGCTTCTCGGGCTTCCT, ActinFw: CCCAAGGGGCGCATTGGCAT, ActinRw: GGGAGGGGGCAGTCAGAGCA.

### Statistical analysis

Results are reported as mean ± SD. Data were normalized versus control. Three independent experiments were analyzed. All values are expressed as mean ± SD. Statistical analysis were performed with Student's T-test for unpaired samples and normally distributed data sets. Statistically significant differences were considered at P < 0.05. (Statview, SAS Institute).

## Discussion

In this study we have demonstrated that (a) CPCs express functional anaphylatoxin receptors, (b) stimulation with C3a or C5a induces the expression of myofibroblast differentiation markers and EndMT-like gene expression, (c) C3a and C5a both increase CPC proliferation and migration and (d) C3a and C5a also induce telomerase activity (NFκB-dependent) and increase telomere maintenance, although not sufficiently to fully preserve telomere length. Taken together these data support a role for anaphylatoxins in CPC regulated cardiac healing and scar formation.

The discovery of endogenous c-kit–positive CPCs has driven a shift in cardiac biology. Work from multiple laboratories (revised in Barile et al. 2007; Laflamme and Murry 2011) have documented the heart’s ability to replace old and dying cells, and propose that this capacity depends on the persistence of a stem cell compartment. The initial interest generated by the identification and isolation of c-kit-positive CPCs was followed by a period of doubt, reflected in studies from different laboratories and accompanying editorials that questioned or neglected issues related to CPC function. These reservations regarding the role of CPCs in cardiac cell turnover are mainly related to the limited nature of myocyte renewal in the human heart (Bergmann et al. 2009). Although multiple studies in animal models have suggested that the adult heart is capable of some cellular turnover (reviewed in Choi et al. 2012), clinical evidence clearly shows that regeneration is inadequate after injury. This evidence supports the hypothesis that endogenous CPC populations probably have a role in cardiac homeostasis other than cardiomyocyte turnover, since the cardiac healing response is very poor. In adult mammals, cardiomyocyte regeneration is insufficient to functionally renew severely injured myocardium and consequently, scar tissue forms. Thus, the evolutionary adapted healing processes that initially benefited cardiac function finally results in a potentially maladaptive response in the long term.

After a myocardial infarction, massive cell infiltration into the myocardium results in fibrosis. MI injury is considered an inflammatory state, characterised by innate immune responses. Whereas it was initially thought that the immune system protects the body against foreign or non-self signals, there is now a body of evidence to suggest that the innate immune system is activated following tissue injury, triggering the release of endogenous ligands (Arslan et al. 2011). Participation of the complement system in myocardial ischemia was first demonstrated in 1971 in a rat MI model (Hill and Ward 1971). Administration of monoclonal antibodies against C5 and C5a as well as the C5a receptor reduced myocardial infarct size in pigs and rodents (Amsterdam et al. 1995; Vakeva et al. 1998; van der Pals et al. 2010; Zhang et al. 2007). However, a study concerning patients either undergoing percutaneous coronary intervention (PCI) or coronary artery bypass grafting (CABG) with pexelizumab (a single-chain fragment of a humanised monoclonal antibody against complement component C5) administration showed a significant reduction in mortality in the former case, but no benefit in patients with acute MI in the latter case (Testa et al. 2008). It is important to note that anaphylatoxin inhibition in these models has been performed in an acute MI phase but that the principal role of anaphylatoxins and their receptors here is to increase leukocyte recruitment to the reperfused myocardium following MI.

Since the complement system is activated in the milieu of tissue injury, the effect of C3a and C5a on CSC migration and proliferation is of specific interest, because these anaphylatoxins may contribute to the mobilisation, recruitment, and proliferation of cells at an injury site, thus facilitating wound healing. Our data support the role of anaphylatoxins in recruiting cells involved in tissue healing: C3a and C5a are able to induce CPC proliferation and migration, which supports the role of anaphylatoxins in the control of tissue healing. Indeed, apart from the known chemotactic role that C3a and C5a play with regard to leukocytes, anaphylatoxins are also chemotactic factors for other cells like mesenchymal stem cells (Schraufstatter et al. 2009) or neural stem cells (Shinjyo et al. 2009). Our data also shows that in addition to this enhancement in proliferation and migration, anaphylatoxins also induce telomerase mRNA and activity. Although addition of C5a significantly increases telomere length compared to controls, and to a lesser degree C3a, telomerase activity is not enough to maintain telomere length compared to the initial state. Interestingly a large body of data has accumulated regarding the capacity of telomerase to support roles other than telomere preservation (reviewed in Martinez and Blasco 2011). One of these roles is the facilitation of cell growth and proliferation. For instance, hTERT overexpression lengthens the proliferative lifespan of human bone marrow stromal cells (Simonsen et al. 2002) being also able to induce hyperplasia and hypertrophy in murine cardiac myocytes (Kohl 2001). Moreover, ectopically expressed hTERT confers resistance to the anti-proliferative effect of transforming growth factor β (TGF-β) in p16-null human mammary epithelial cells (Stampfer et al. 2001). In addition, telomerase activation in human mammary epithelial cells coincides with the stimulation of a cellular mitogenic program (Smith et al. 2003), indicating that telomerase may affect epithelial cell proliferation not only by stabilising telomeres, but also by affecting the expression of growth-promoting genes. There is also data that supports an extra-telomeric role for telomerase in protection against oxidative stress; in this regard Schraufstatter et al. (2009) demonstrated that C3a and C5a protect MSCs from oxidative damage. This can be translated into an *in vivo* scenario, where one would expect that the anaphylatoxins would recruit healing cells to areas of tissue injury, where these cells would encounter the production of oxidants due to neutrophil recruitment and reperfusion injury. Therefore all these data support a role for anaphylatoxins in promoting the recruitment of healing cells to areas of injury in order to restore tissue homeostasis. Telomerase activity would not only improve cell proliferation and survival but also protect cells against the hostile environment – providing protection from apoptosis, oxidative stress, and DNA damage.

Injured ischemic myocardium progresses to necrosis and subsequent healing; the local response to irreversible injury is the formation of granulation tissue, the accumulation of collagen, and resultant replacement fibrous scar (Sun et al. 2002). Although clinical and biological data shows that the heart has a very low endogenous capacity for regeneration when severe damage is inflicted, multiple endogenous putative cardiac stem cell or progenitor cell populations have been identified and isolated. Markers, traditionally associated with blood, bone marrow or pluripotent stem cells, have been used by several independent groups to identify these cells in adult or postnatal hearts in humans and other mammalian species (Smith et al. 2008). We have worked with a population (CPCs), as described by Messina et al. (Messina et al. 2004), this population expresses the cardiac markers, Nkx2.5, Gata4, Cx43 and Mef2c (Figure 1B) and therefore had a putative cardiogenic potential. Thus our first task was to understand why, if these cells have the potential to differentiate in cardiomyocytes, physiological cardiac healing usually results as a fibrotic scar and shows very low levels of cardiomyocyte replacement. We investigated the potential of these cells to differentiate to all three lineages described, and facilitated differentiation by using 10% FBS to improve myofibroblast differentiation, and EGM on Matrigel to increase endothelial lineage differentiation. Interestingly anaphylatoxins repress Gata4 expression and increase the expression of some early cardiac markers (Nkx2.5, Tbx3, Tbx5, Actc1) in CPCs. Endothelial cell (EC) lineage differentiation was clearly improved by culture on Matrigel using EGM media and in these conditions, stimulation with C3a and C5a anaphylatoxins reduced endothelial gene expression. In agreement with this data, immunofluorescence also showed the absence of ECs in the presence of anaphylatoxins. Inhibition of EC differentiation is paralleled by induction of Twist and Snail genes together with Fsp1 expression. Fsp1 is one of the markers of fibroblast formation and is a cytoskeletal protein belonging to the calmodulin-S100-troponin C superfamily of intracellular calcium binding proteins associated with cytoskeletal fibres, cell motility, and mesenchymal phenotype (Zimmer et al. 1995). Therefore the expression of Snail1 and Twist1 genes, together with the significant increase in Fsp1 expression, indicated that an endMT-like differentiation event might be occurring in CPCs. In addition, anaphylatoxins induce the expression of an array of genes associated with myofibroblasts that probably abolishes their role in regeneration but hints at their relevance in the cardiac healing process that follows MI. In this scenario, complement anaphylatoxins would activate migration of CPCs to the damaged area, induce their proliferation and finally, upon persistence of local signalling, would promote their differentiation into myofibroblast cells that would be able to fill and quickly repair the damaged heart area, preventing further cardiac complications.

Gata4 has been proved to be a critical early factor in cardiogenesis, it lays upstream of Tbx5, Nkx2.5, and Act1 in the cardiogenic transcription network, and acts together with Baf60c to generate a chromatin state competent for cardiogenic differentiation (Takeuchi and Bruneau 2009). Therefore C3a and C5a may block cardiomyocyte differentiation by repressing Gata4 expression. The cardiogenic factors Nkx2.5, Actc1 and specially Tbx3 and Tbx5 may have roles other than their cardiogenic ones, including modulating the response of CPCs to complement anaphylatoxins, for instance they might be involved in the C3a/C5a dependent myofibroblast differentiation process. It is known that several mesodermal cells (cardiomyocytes and myofibroblasts) share parts of their transcriptional differentiation networks, supporting the existence of a common myocardial and smooth muscle cell precursor in the developing embryo. This is consistent with *in vivo* studies, which show the co-expression of numerous smooth muscle genes in myocardial progenitor cells (Wu et al. 2006). Therefore Tbx3 and Tbx5 would be common to both cell types, and Gata4 levels would be critical in modulating the fate decision of progenitor cells. Interestingly it has been proposed that Gata4 expression can play a role in the developmental regulation of cardiac fibroblasts and has a function in the maintenance of cardiac-resident progenitors (Jankowski 2009). This raises the hypothesis that C3a and C5a are factors that affect CPC fate by switching the balance of different lineage specific factors: they shut down the expression of endothelial and cardiac factors and induce the expression of myofibroblast factors (TCF21, SM22α and Myocardin), irreversibly pushing CPCs towards the myofibroblast fate.

Taken together, these findings shed some light onto what have been described as endogenous cardiac stem cells and some mechanistic insight about the limited potential of CPCs to generate new cardiomyocytes after cardiac injury. We can hypothesize that anaphylatoxin release at early time after cardiac injury is able to increase CPC numbers and to promote migration towards the site of injury. This signal also increases CPC potential to differentiate into myofibroblast lineages that would participate in scar formation. This situation gives an advantage to cardiac tissue promoting a fast healing in MI. Differentiation toward myofibroblast in CPCs would make available a higher number of myofibroblasts to contribute to other sources of myofribroblasts (Krenning et al. 2010) during the resolution stage of MI. This situation is different from physiological cardiac cell turnover. During life possibly other signals in absence of inflammatory signals will permit cardiomyocyte differentiation to replace exhausted cells. Interestingly recent evidence reported by Jianqin Ye et al. (2012) have shown that there is a significant increase in the proliferative capacity of CS-forming cells isolated from the “middle aged” heart following acute MI resulting in a significant rise in the number of CSs in vitro and this increase is most pronounced within the first week post-MI. In addition they show that show for the first time that the CS cells obtained from 1-week post-MI hearts engraft in ischemic myocardium and restore cardiac function at 25 days post-injection in vivo. However, we did not find evidence for differentiation of these cells into mature cardiomyocytes or new vessels althouhgt promoted angiogenesis in vivo. Their data suggest that early signals that happens after MI would commit these cells to a more healing phenotype instead of regenerative fate.

To heal the heart adding new cardiomyocyte probably is a more delicate and time-consuming situation that mammalian wounded heart cannot go through in a wild environment. Thus the signals involved in cardiac healing have been selected to permit a fast healing situation increasing myofibroblast differentiation but in other hand this impairs cardiomyocyte renewal. Deeper understanding of these mechanisms would help to improve wounded heart regeneration.
